# The Size Effects of Modified Nano-Silica on the Physical Properties of Resorcinol-Poly(acrylamide-co-2-acrylamido-2-methylpropanesulfonic acid) Gels in Harsh Reservoir Conditions

**DOI:** 10.3390/gels11100769

**Published:** 2025-09-24

**Authors:** Xun Zhong, Yuxuan Yang, Jiating Chen, Yudan Dong, Sheng Lei, Hui Zhao, Hong He, Lifeng Chen

**Affiliations:** 1 College of Petroleum Engineering, Yangtze University, Wuhan 430100, China; yangyuxuan0000@163.com (Y.Y.); 18653262028@163.com (Y.D.); zhaohui@yangtzeu.edu.cn (H.Z.); hehong1103@163.com (H.H.); lyj505523522@126.com (L.C.); 2 Key Laboratory of Drilling and Production Engineering for Oil and Gas, Wuhan 430100, China; 3 Shunan Gas Mine, Southwest Oil and Gas Field Branch, China National Petroleum Corporation, Luzhou 646000, China; chenjiating@petrochina.com.cn; 4 Western Research Institute, Yangtze University, Karamay 834000, China

**Keywords:** modified nano-silica, harsh reservoir conditions, gel strength, long-time stability, overfilling

## Abstract

Nano-silica is widely used to enhance gel properties, but its size, concentrations, and aggregation behaviors all matter. The influencing rules of these factors remain unclear especially in harsh reservoir conditions. This study presented a comprehensive investigation into the gelation, rheological, and plugging properties of phenolic polymer gels reinforced by modified nano-silica (GSNP) of different sizes and concentrations in harsh reservoir conditions. Specifically, the nano-silica was modified with a highly soluble silane, and gel properties were evaluated through rheological, differential scanning calorimetry (DSC), and sandpack flooding tests. The results showed that the incorporation of GSNP prolonged the gelation time, enhanced gel strength, and improved stability, allowing the gelation solution to enter deeper into the formation while maintaining long-time effectiveness. The optimal gel system was obtained with 0.4 wt.% GSNP-30, under which condition the storage modulus increased by approximately 14 times, and the content of non-freezable bound water more than doubled. This system exhibited plugging efficiency exceeding 80% in formations with permeabilities ranging from 1000 to 6000 millidarcy and enhanced the oil recovery factor by over 25%. The reinforcement mechanisms were attributed to the adsorption of GSNP onto polymer chains and its role in filling the gel matrix, which enhanced polymer hydrophilicity, suppressed polymer aggregation/curling, prevented ion penetration, and promoted the formation of a more uniform gel network. Careful optimization of nanoparticle size and concentration was essential to avoid the detrimental effects due to nanoparticle overfilling and aggregation. The novelty of this study lies in the practicable formulation of thermal and salt-tolerant gel systems with facile modified nano-silica of varying sizes and the systematic study of size and concentration effects. These findings offer practical guidance for tailoring nanoparticle parameters to cater for high-temperature and high-salinity reservoir conditions.

## 1. Introduction

Profile control and water shutoff techniques are essential to mitigate the prevalent problems of early water breakthrough, excessive water production, and strong reservoir heterogeneity. A thorough understanding of reservoir conditions and economic considerations is the premise of technology design and formula optimization. Polymer gel water control technology dates back to the 1970s~1980s and after decades of development has evolved into one of the widely used and cost-effective water management techniques [1,2,3,4]. The gelation time and strength of polymer gels can be readily adjusted to cater for the specific requirements of different reservoirs. By blocking thief zones and diverting injected water, highly viscoelastic polymer gels are capable of mitigating interlayer contradiction and improving the overall water sweep efficiency [5,6,7].

A major challenge encountered when implementing polymer gel for field applications is maintaining gel stability in reservoirs, particularly in high-temperature and high-salinity conditions. Elevated temperatures accelerate molecule motion and shorten gelation time, making polymer gel incapable of penetrating deep into the formation. Moreover, chain scission in polymers and crosslinkers, along with polymer hydrolysis occurring in elevated temperature conditions, would significantly reduce gel strength [8]. High salinity intensifies cation–polymer interactions (e.g., Ca^2+^/Mg^2+^ with polymer–COO^−^), reducing gel hydrophilicity and water-holding capacity, which compromises network integrity and plugging performance [9,10]. Two common strategies to enhance gel stability in harsh conditions are developing novel thermal and salt-tolerant polymers and incorporating stabilizers. However, designing and scaling up novel polymers is usually challenging, time-consuming, and costly [11,12]. As a result, the incorporation of stabilizers, especially nano- and micron-sized particles, becomes a hot trend [13,14,15,16,17]. For instance, Almeida et al. [18] compared the impacts of graphene oxide, oxidized carbon nanotubes, and titania nanoparticles on polyacrylamide/polyethyleneimine (PAM/PEI) gel systems at 70 °C and 85.0 g/L salinity. All three nanomaterials improved gel properties and the syneresis percentages were reduced by over 70.0% with titania. Similar improvements were reported by Xu et al. [19]. Li et al. [20] developed a gel system that was stable at 130 °C, 230.72 g/L salinity for over 120 days, with syneresis rate below 2.5% by adding 0.5 wt.% polyamide nylon fibers to a PAM/PEI gel. The plugging rate was 94.0%. Shi et al. [21] incorporated 3.0 g/L micro-sized graphite powder into a polyacrylamide gel and prepared a strong gel with an elastic modulus of 400 Pa to address the excessive water production issue in a fractured low-permeability reservoir. Ilavya et al. [22] incorporated modified nano-graphene oxide into a phenolic gel to enhance gel framework resilience and shorten gelation time. Lv et al. [23] prepared a long-term stable gel at 130 °C and 200.0 g/L salinity by integrating modified nano-graphite into a PAM system. The composite gel demonstrated good shear resistance, strong erosion resistance, and excellent plugging capability.

Nano-silica is cost-effective, widely accessible, and can be easily fabricated and functionalized, making it highly suitable for engineering purposes. In recent decades, nano-silica-reinforced polymer gel systems have been extensively investigated [24,25,26,27], and the summary of the recent progress is presented in Table 1. Telin et al. [28] reported that both hydrophilic and hydrophobic nano-silica could enhance gel properties. The addition of hydrophilic nano-silica increased the elastic modulus of the gel by over 55% and the produced gel remained stable for more than one year. Liu et al. [29] reported that incorporating 3.0 wt.% nano-silica increased the maximum temperature tolerance of a phenolic gel system by 12.8%, attributed to increased bound water content and better water-holding capacity. Furthermore, nano-silica has been proposed as an effective remedy to counteract gel embrittlement caused by retarders such as ammonium chloride [30,31]. However, nanoparticles are prone to aggregate especially in high-temperature and high-salinity conditions in pursuit of a more stable state. Nanoparticle agglomeration is detrimental to gel properties [32,33] and common strategies to enhance the applicability of nano-silica in harsh reservoir conditions include increasing nanoparticle dosage and conducting nanoparticle surface modification [34,35,36]. For instance, Wang et al. [37] developed a low-cost, long-term stable phenolic gel containing 1.0 wt.% nano-silica suitable for 130 °C and 41.529 g/L salinity. Similarly, Mandal et al. [38] prepared several composite gels that were stable at 120 °C and 60.0 g/L salinity for over two years with plugging efficiencies exceeding 90% by increasing nano-silica dosage to 1.0~5.0 wt.%. The temperature tolerance increased by 5 °C and 64 °C for low- and high-molecular-weight polymer gels, respectively. Zareie et al. [39] prepared a nano-hybrid gel capable of withstanding 211.688 g/L salinity at 90 °C with 9.0 wt.% nano-silica. Huang et al. [40] functionalized silica with hydro sulfonyl and oleic groups and formulated a composite gel applicable at 17.0 g/L salinity. Through diazotization and oxidation reactions at 0~5 °C, Qian et al. [41] synthesized phenolic-hydroxyl-group-modified nano-silica and subsequently developed a nano-hybrid phenolic gel that was stable at temperatures below 140 °C for 60 days with a syneresis rate below 10% by incorporating 0.15~0.2 wt.% nano-silica. Nonetheless, the approaches often involve high cost or demanding synthesis conditions.

To the best of our knowledge, there are limited studies that investigate the size effects of nano-silica on the gelation and rheological properties of nanocomposite gels, and even fewer studies have elucidated the underlying mechanisms governing polymer–crosslinker–nanoparticle interactions in high-temperature and high-salinity conditions. In this study, poly(acrylamide-co-2-acrylamido-2-methylpropanesulfonic acid) (PAM/AMPS copolymer) gels crosslinked with hexamethylenetetramine (HMTA) and resorcinol are developed, and the impacts of modified nano-silica (GSNP) size (10~100 nm) and concentrations (0~1.0 wt.%) on the properties of nanocomposite gels are evaluated. A comprehensive analysis is conducted to assess the gelation, rheological, structural, and plugging properties, aiming to reveal the mechanisms by which modified nano-silica enhances gel properties in harsh reservoir conditions. This work seeks to provide insights into the role of nanoparticle assembly within gel networks when exposed to high temperature and high salinity.

The novelty of this study lies in the practicable formulation of thermal and salt-tolerant gel systems with facile modified nano-silica of varying sizes and the systematic study of size and concentration effects on the gelation, structural, and plugging properties of nanocomposite gels in harsh reservoir conditions. Moreover, the possible functional mechanisms of nano-silica of different sizes and concentrations are proposed and compared.

## 2. Results and Discussions

### 2.1. Dispersity of GSNP

**Figure 1:**
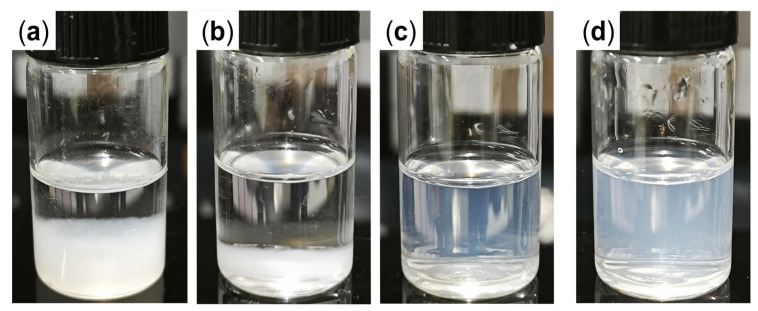
Dispersity of nano-silica. Bare nano-silica in (**a**) synthetic brine and (**b**) polymer solution; and GSNP in (**c**) synthetic brine and (**d**) polymer solution.

Bare nano-silica undergoes severe aggregation and significant precipitation within 4 h at 90 °C when dispersed in synthetic brine (Figure 1a), primarily due to strong interactions between surface silanol groups and Ca^2+^/Mg^2+^, as well as compression of the electric double layer [49,50,51]. Increasing the base fluid viscosity by using a 0.5 wt.% PAM-AMPS-35 polymer solution as dispersant does not markedly improve the dispersing stability of bare nano-silica (Figure 1b), suggesting that polymer alone can hardly counteract the adverse effects of high cation concentrations at elevated temperatures.

**Figure 2:**
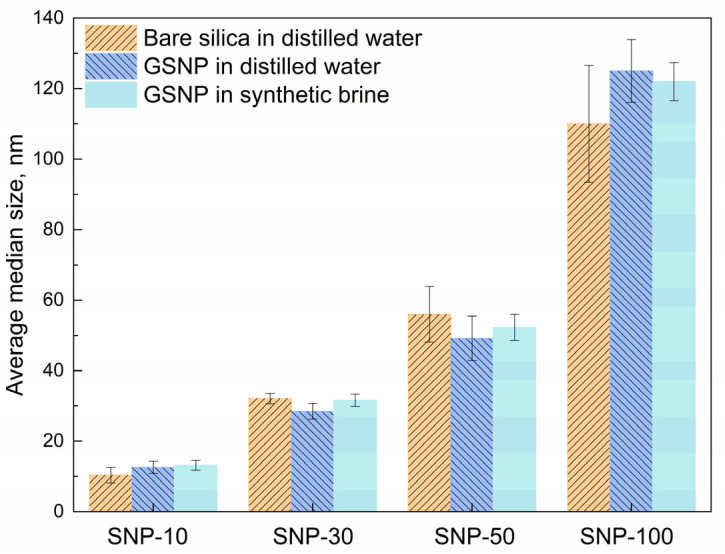
Comparison of average median size of nano-silica in different dispersing media.

In contrast, surface-modified nano-silica (GSNP) exhibits significantly improved dispersing stability. A transparent solution is obtained when 0.1 wt.% GSNP is dispersed in synthetic brine (Figure 1c). As shown in Figure 2 and Table S1, the average median size of GSNP in distilled water remains barely unchanged after modification. Moreover, increasing the salinity to ~6.7 wt.% has no significant effects on GSNP size. Polymers can improve nanoparticle dispersity by increasing the viscosity of the dispersing medium. However, adsorption of polymer chains onto the particle surface may also induce bridging effects and intensify particle agglomeration. To test the stability of GSNP in polymer solution, a GSNP-PAM/AMPS-35 mixed system is prepared and aged at 90 °C. The system remains homogeneous without noticeable precipitation for 120 h, suggesting that GSNP is a promising stabilizer for polymer gel systems in harsh reservoir conditions. The excellent compatibility of GSNP with both synthetic brine and polymer solutions is mainly attributed to the strong steric repulsion between GSNPs imparted by the grafted ligands [52].

### 2.2. Effects of GSNP on Gelation Properties

#### 2.2.1. Gelation Time

**Figure 3:**
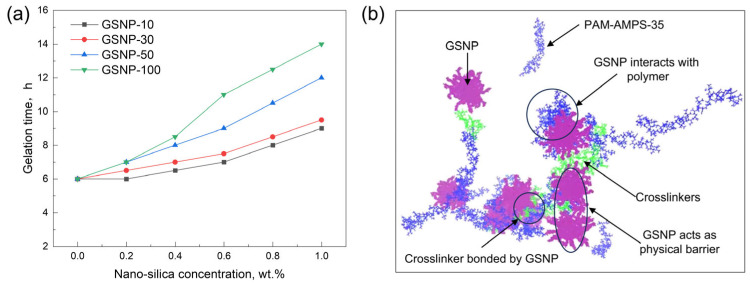
Impacts of GSNP concentration and size on gelation time (**a**) and representative interactions between polymers, GSNP, and crosslinkers (**b**).

The crosslinking process of a phenolic gel mainly includes three stages, thermal decomposition of HMTA, formation of hybrid phenolic polymers, and formation of a robust gel network. When nano-silica is added, the initial viscosity of all gel solutions remains around ~75 mPa·s, indicating that GSNP incorporation has minimal impacts on the flow behavior of the gelation solution. As the reaction proceeds, viscosity increases, and gel starts to form. The gelation time gradually increases with increasing nanoparticle concentrations, as shown in Figure 3. The impacts are most pronounced with GSNP-100, while GSNP-10 and GSNP-30 are less significant. With the addition of 1.0 wt.% GSNP-100, gelation time prolongs to 14 h from 6 h, which enables better transportation of the gelation solution to the deeper formations. This delay is likely because GSNP can act as a physical barrier, interacting with polymer chains, reducing the concentration of crosslinkers (Figure 3b), and thereby slowing polymer–crosslinker interactions and gel network formation [34]. Additionally, GSNP may interact with the intermediate products (e.g., hydroxymethyl groups) via hydrogen bonding or electrostatic interactions, thus reducing the activity of reactants. Generally, larger nanoparticles impose greater steric hindrance, thus leading to more noticeable prolongation of gelation time.

#### 2.2.2. Gel Strength

**Figure 4:**
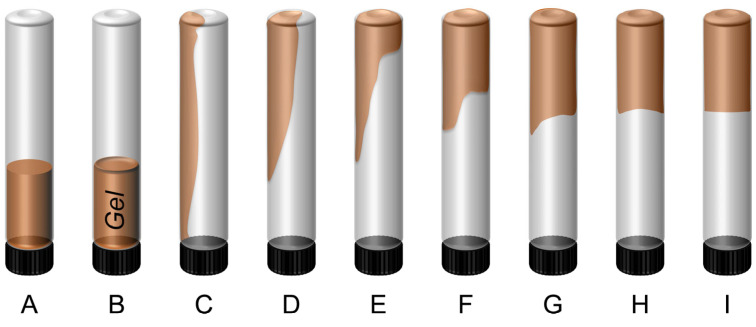
Sydansk gel strength codes.

Based on gel deformability, gel strength is classified into nine grades (A~I), as shown in Figure 4. Grade “A” suggests no detachable gel formation, and the viscosity is similar to that of the initial polymer solution. Meanwhile, Grade “I” corresponds to a rigid gel showing no detectable surface deformation upon inversion [26,53]. Without nanoparticles, the resulting gel is weak and flowable (Grade “C”), with most gel flowing to the bottle cap when inverted. Incorporation of 0.1~1.0 wt.% GSNP of various sizes reduces deformability and increases gel strength. With 0.4~0.6 wt.% GSNP-30 or GSNP-50, gel strength reaches a maximum (Grade “F”), and a weak gel system is transformed into a strong one. A further increase in GSNP concentration results in enhanced flowability and reduced gel strength (Table 2).

#### 2.2.3. Gel Stability

**Figure 5:**
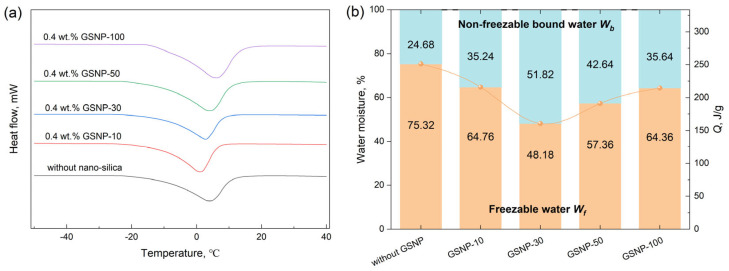
(**a**) DSC curves and (**b**) non-freezable bound water contents of gel samples with 0.4 wt.% GSNP of different sizes.

**Figure 6:**
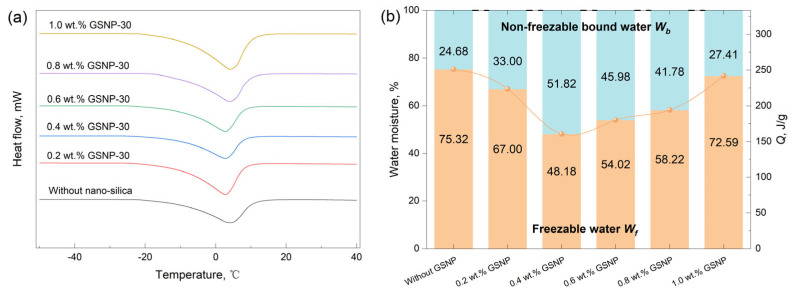
(**a**) DSC curves and (**b**) non-freezable bound water contents of gel samples with GSNP-30 of different concentrations.

Water in gel systems is categorized into two types, non-freezable bound water and freezable water [54]. Non-freezable bound water refers to the water that remains in a liquid state even when the temperature drops below its normal freezable point due to the strong interactions between water molecules and other components, and its content is a key indicator to evaluate the water-holding capacity and the long-time stability of a gel system [55]. Figure 5 and Figure 6 compare the mass fractions of non-freezable bound water in different GSNP–composite gel systems after being aged at 90 °C for 10 days. All DSC curves demonstrate an endothermic peak near 0 °C due to the melting of freezable water. A proper addition of GSNP effectively suppresses gel syneresis and generally increases the proportion of non-freezable bound water. The effects of GSNP-10 and GSNP-100 are less pronounced than those of GSNP-30 and GSNP-50, likely due to the excessively strong interactions between small-sized nanoparticles or the relatively weak integration of larger particles within the polymer matrix. Additionally, the concentration of GSNP plays a crucial role. Too many nanoparticles may lead the gel network to collapse, while too few are insufficient to provide structural support. The proportion of non-freezable bound water increases by nearly 1.1 times, peaking at 51.82% when 0.4 wt.% GSNP-30 is incorporated, compared to that of 24.68% in the conventional gel system. Non-freezable bound water is tightly bonded with the gel network and is less prone to separation. A higher proportion indicates greater resistance to dehydration and improved stability.

### 2.3. Effects of GSNP on Rheological Properties

#### 2.3.1. Storage Modulus

**Figure 7:**
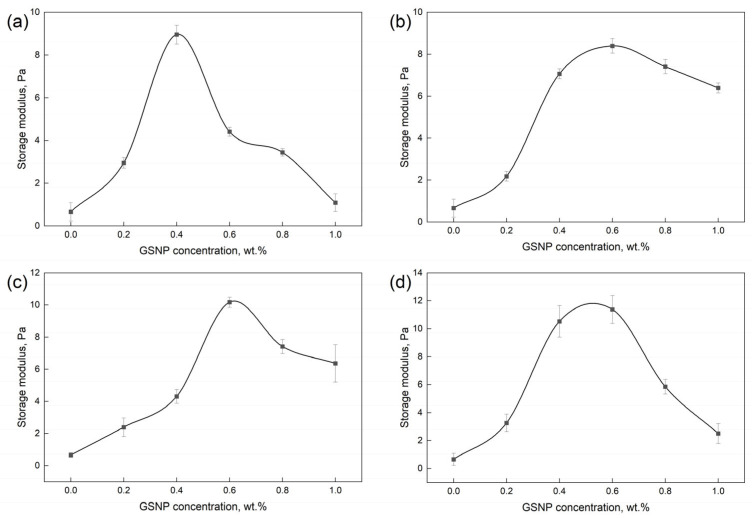
Impacts of GSNP concentration on the storage modulus of nanocomposite gels. (**a**) GSNP-10, (**b**) GSNP-30, (**c**) GSNP-50, and (**d**) GSNP-100.

The storage modulus (G’) reflects the energy stored in the system and represents the elastic strength of gels. A higher G’ indicates a greater ability to withstand stress and a stronger gel structure. The storage modulus of polymer gels strengthened by GSNP of varying sizes at different oscillation frequencies is presented in Figure S1, and the linear viscoelastic region (LVR) storage modulus is shown in Figure 7. Adding a proper amount of GSNP significantly enhances gel strength, and the highest strength is achieved by incorporating 0.4~0.6 wt.% GSNP due to increased interactions with the polymer matrix and additional crosslinking sites. However, excessive GSNP (>0.6 wt.%) is detrimental to gel strength, likely due to nanoparticle collision and aggregation and decreased dispersity at high concentrations. The adverse impacts are less noticeable with medium-sized GSNP (GSNP-30 and GSNP-50) compared to large-sized (GSNP-100) or small-sized (GSNP-10) particles.

#### 2.3.2. Loss Factor

Polymer gels exhibit both elastic and viscous properties, and their long-term stability can be characterized by the loss factor, as defined in Equation (1). Generally, a lower loss factor corresponds to higher stability [26].

(1) tanδ=G″G′

where *tanδ* stands for the loss factor, $G^{\prime \prime }$ and $G^{\prime }$ are loss modulus and storage modulus, respectively.

**Figure 8:**
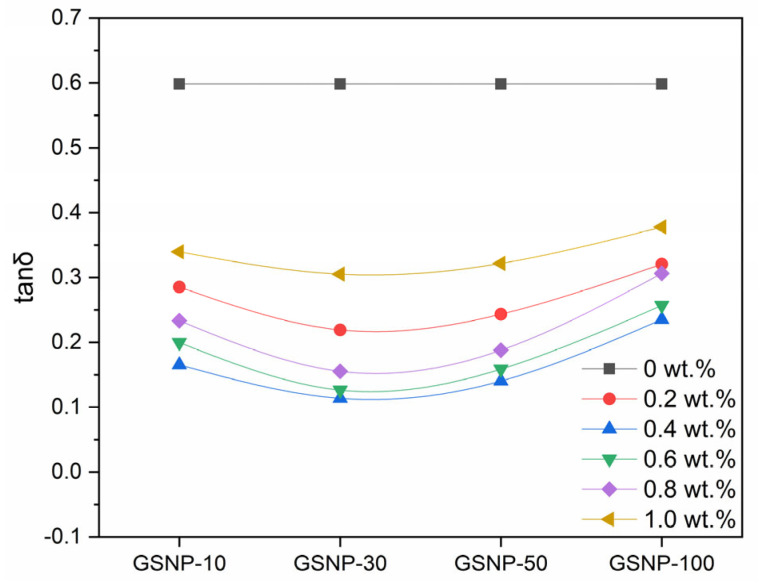
Impacts of GSNP concentration and size on the loss factor of nanocomposite gels.

As shown in Figure 8, across all the tested nano-silica sizes and concentrations, the loss factor remains below 1.0, confirming solid-like behavior of the gel samples. GSNP-30 composite gels demonstrate more solid-like features. The loss factor decreases first and then increases with increasing GSNP concentration, with the most stable behavior occurring at around 0.4 wt.%. Beyond this optimal dosage, excessive nanoparticles may hinder the crosslinking reaction and promote a shift toward viscous behaviors.

### 2.4. Effects of GSNP on Plugging Efficiency and EOR Performance

#### 2.4.1. Plugging Efficiency

**Figure 9:**
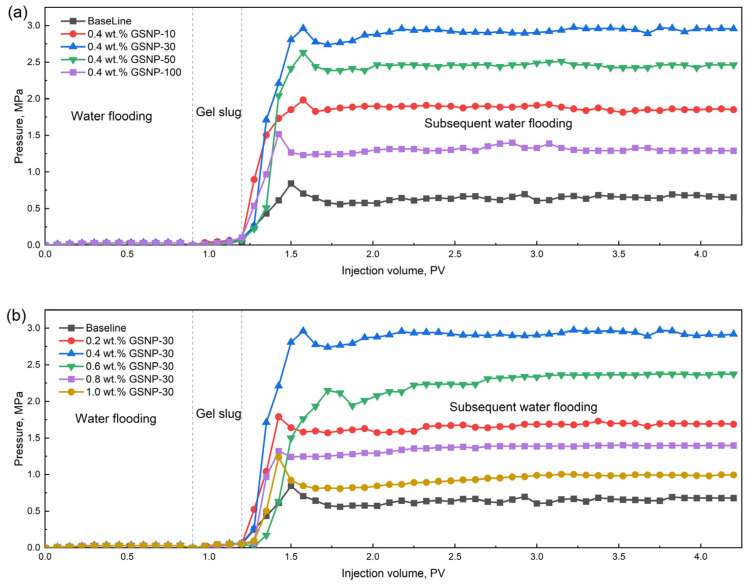
Impacts of GSNP concentration (**a**) and size (**b**) on plugging performance.

The plugging efficiency of various gel systems with a slug size of 0.3 PV is shown in Figure 9. For sandpacks with permeability around 1000 millidarcy, the injection pressure of the gelation solution is approximately 0.05 MPa and the plugging rates are generally above 95%. The breakthrough pressure of the optimal gel system containing 0.4 wt.% GSNP-30 reaches about 2.9 MPa, indicating good injectivity and effective plugging performance, confirming its suitability for such permeability conditions. Furthermore, the equilibrium pressure during subsequent water flooding (with a slug size of 3 PV) manifests that the composite gels demonstrate excellent resistance to fluid flow. Among the different nanoparticles tested, gels reinforced with GSNP-30 and GSNP-50 exhibit more satisfying plugging performance compared to those with GSNP-10 or GSNP-100. The results are consistent with the trends observed in Section 2.2 and Section 2.3.

**Figure 10:**
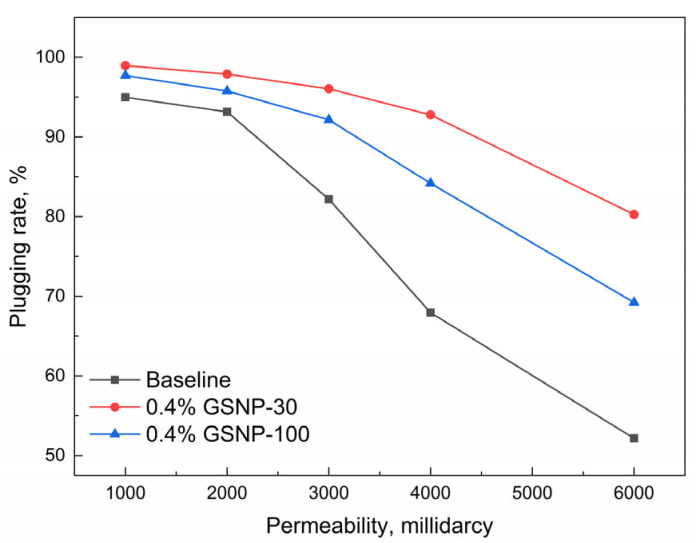
Impacts of permeability on the plugging performance of gel systems.

The compatibility of GSNP-30- and GSNP-100-reinforced gels with formations of different permeabilities is further illustrated in Figure 10. The plugging rate decreases as permeability increases. But the GSNP-30-reinforced gel consistently outperforms both the conventional polymer gel and the GSNP-100-reinforced gel. When the permeability is 3000 millidarcy, the plugging rates of GSNP-reinforced gels exceed 90%, compared to 82.17% of the conventional polymer gel. When permeability increases to 6000 millidarcy, the plugging rate of the conventional gel declines to around 50%, while the GSNP-30-reinforced gel maintains a plugging rate above 80%.

#### 2.4.2. EOR Performance

**Figure 11:**
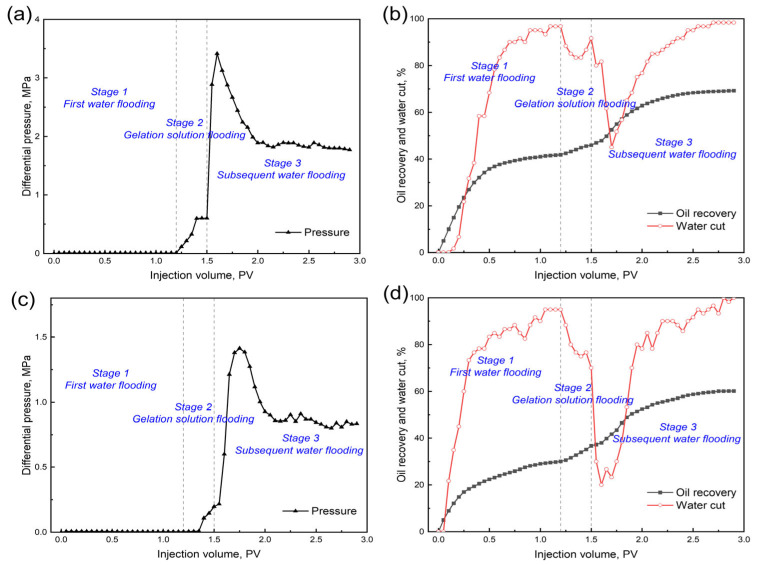
Differential pressure, oil recovery, and water-cut curves at different permeability ratios. (**a**) and (**b**) k1/k2 = 2:1; (**c**) and (**d**) k1/k2 = 6:1.

**Figure 12:**
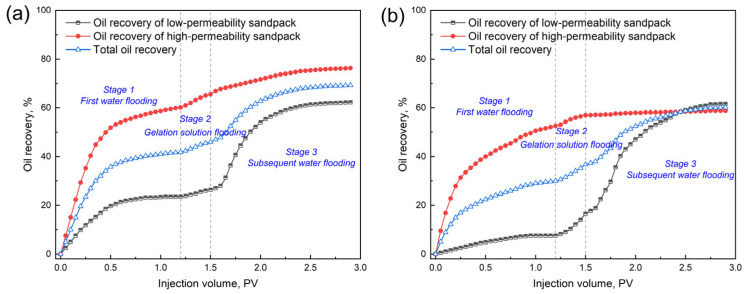
The recovery factors of different layers at different permeability ratios. (**a**) k1/k2 = 2:1; (**b**) k1/k2 = 6:1.

The EOR performance of the 0.4 wt.% GSNP-30-reinforced gel is evaluated in heterogeneous formations with permeability ratios of 2:1 (Set 1) and 6:1 (Set 2), with a gel slug size of 0.3 PV. The low-permeability layer has a permeability of 1000 millidarcy, while the high-permeability layers are 2000 millidarcy and 6000 millidarcy, respectively. Before gel treatment, over 80% of the injected water preferentially flows through the high-permeability zone, leaving the low-permeability layer largely unswept. The differential pressure, oil recovery, and water cut at different stages (e.g., first water flooding stage, gelation solution flooding stage, and subsequent water flooding stage) are monitored throughout the process, as presented in Figure 11 and Figure 12. First water flooding recovers 41.83% and 30.04% of the original oil in place for Sets 1 and 2, respectively. In Set 1 (permeability ratio is 2), the recovery factors are 23.5% and 60.17% for the low- and high-permeability layers, respectively, compared to those of 7.5% and 52.58% in Set 2 (permeability ratio is 6). During gelation solution injection, the differential pressure in Set 1 is significantly higher and more fluctuating due to narrower pore throats and higher flowing resistance. After gel formation and aging, subsequent water flooding results in additional total oil recovery increments of 27.42% and 30.13% for Sets 1 and 2, respectively, indicating the effectiveness of the nanocomposite gel in mitigating heterogeneity and improving overall sweep efficiency.

### 2.5. Mechanism Analysis

Polymer gels lose thermal stability due to the rupture and/or hydrolysis of polymer chains and the breakage of crosslinker chains [8]. In this section, the interactions among nano-silica, polymer, and crosslinkers are discussed based on the experimental observations to reveal the possible underlying mechanisms through which nano-silica enhances both the strength and the water-holding capacity of polymer gels.

#### 2.5.1. Gel Strength Improvement

**Figure 13:**
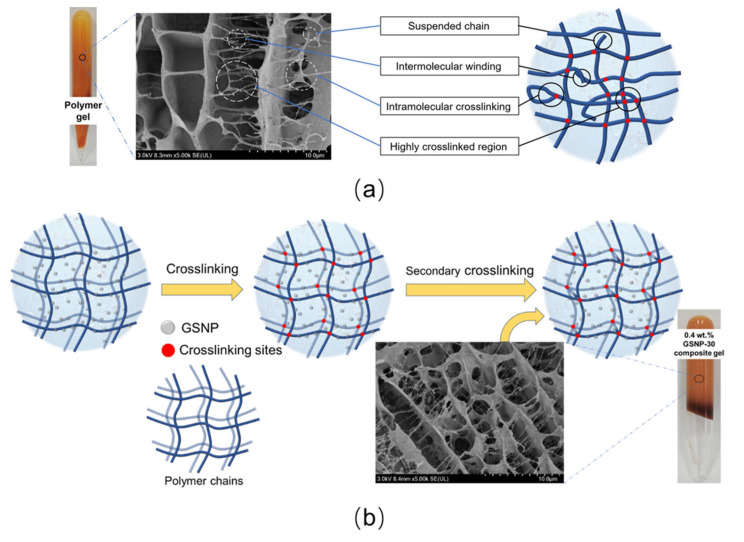
Schematic diagram of gel network with or without nano-silica. (**a**) Without nano-silica; (**b**) with a proper amount of nano-silica.

Polymer gels without nanoparticles generally demonstrate a non-uniform structure characterized by randomly distributed highly crosslinked regions, suspended chains, and intramolecular crosslinking [37,49], as shown in Figure 13a. In high-temperature and high-salinity conditions, the network grids become even more irregular due to the strong interactions between polymer hydrophilic groups (e.g., carboxyl groups) and the cations (e.g., Ca^2+^ and Mg^2+^), resulting in polymer precipitation and disruption of the polymer matrix continuity. The incorporated GSNPs would adsorb onto polymer chains via hydrogen bonding or electrostatic attractions [18,55], thereby (1) enhancing polymer hydrophilicity and oxidation resistance by bonding with free amides and carboxylic acids, (2) acting as physic barriers to inhibit polymer bridging and aggregation, (3) restraining ion penetration and mitigating salting-out effects on crosslinking reactions, and (4) serving as nucleation sites to facilitate the growth of a more uniform polymer network to better support the gel skeleton (Figure 13b) and to increase the resistance of polymer chains to relaxation at high temperatures. As a result, the overall gel strength is enhanced.

**Figure 14:**
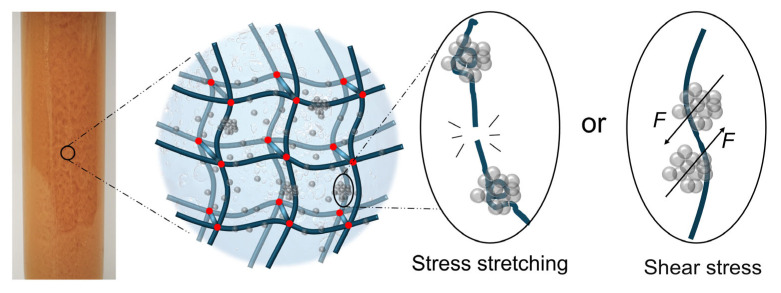
The integrity of gel network being destroyed by stress concentration when in the presence of excessive nano-silica.

The dosage of nanoparticles is also critical. A proper amount of GSNP enhances gel properties, whereas excess GSNP can be detrimental. When most crosslinking sites are occupied by GSNPs, the available crosslinkable sites for polymer, HMTA, and resorcinol are reduced, leading to low crosslinking density and the failure of forming a complete, ordered elastic network. Moreover, higher GSNP concentration increases the likelihood of particle collision and aggregation. Aggregation not only increases particle size and reduces reinforcement efficiency but also introduces defects and concentrated stress points within the gel network, compromising matrix integrity and diminishing gel strength [32,33]. The formation of concentrated stress points is likely due to (1) polymer curling and decreases in the elasticity of polymer chains that are bound by the crosslinkers and (2) the dislocation of local polymer chains under shear action by nanoparticle aggregates, as depicted in Figure 14.

#### 2.5.2. Water-Holding Capacity Improvement

In the absence of GSNP, highly crosslinked regions are noticeable in conventional polymer gel in high-temperature and high-salinity conditions due to severe polymer entanglement and aggregation and limited interactions between polymers and water molecules. These highly crosslinked regions increase local stiffness and reduce crosslinking homogeneity. The narrow grid spacing between crosslinked junctions further weakens the elastic deformation and water-holding capacity [46].

Incorporating a proper amount of nano-silica enhances polymer hydrophilicity and relieves polymer entanglement, resulting in a more uniform network. Some freezable water may turn into non-freezable bound water due to strong interactions among polymers, rigid nano-silica, and water molecules. Additionally, adsorbed nano-silica would act as physical barriers, suppressing chelation between polymers and cations (e.g., Ca^2+^ and Mg^2+^) and thereby effectively slowing down gel dehydration. However, increases in particle size or reductions in effective specific surface area would reduce the effectiveness of GSNP to enhance water-holding capacity.

**Figure 15:**
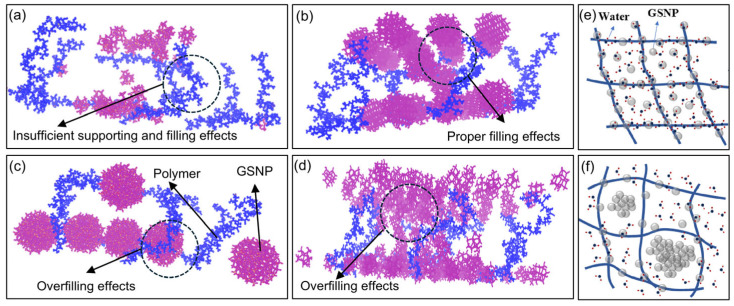
The filling effects of GSNP with different sizes and concentrations. (**a**) Insufficient supporting and filling effects of small-sized GSNP, (**b**,**e**) proper filling effects of medium-sized GSNP. (**c**) Overfilling effects of large-sized GSNP, (**d**,**f**) overfilling effects of excessive GSNP. Color identification: GSNP, red; Gel network, blue.

Small nanoparticles with a larger specific surface area and higher reactivity are more prone to aggregation, which accelerates the breakdown of polymer matrix and gel structure. For a fixed concentration, larger nano-silica particles are fewer in number, resulting in relatively weaker water-holding capacity. Additionally, the filling effect of GSNP also plays a crucial role (Figure 15). Proper filling effects of medium-sized GSNP are beneficial to maintain the regular structure of gel networks. Meanwhile, insufficient supporting and filling effects of small-sized GSNP or overfilling effects by large-sized GSNP would lead to gel network deformation and polymer chain curling, thus weakening the interactions between polymer and water molecules. Additionally, increases in particle size or concentration would decrease the effective free space for water molecules. Therefore, optimizing nanoparticle size and concentration to maximize the proportion of non-freezable bound water is essential for enhancing the water-holding capacity of polymer gels.

## 3. Conclusions

In this study, the gelation, structural, and plugging properties of phenolic polymer gels enhanced by modified nano-silica (GSNP) are comprehensively investigated in high-temperature and high-salinity conditions, with emphasis on the impacts of nanoparticle size and concentration. The following conclusions can be drawn from the experimental results.

(1) The strengthening effect of GSNP is highly dependent on particle size and concentration. The optimal gel system suitable for harsh reservoir conditions is obtained with the incorporation 0.4 wt.% GSNP-30 into a base formulation containing 0.5 wt.% PAM-AMPS-35, 0.35 wt.% HMTA, and 0.25 wt.% resorcinol.
(2) With the addition of 0.4 wt.% GSNP-30, the storage modulus of the gel increases by approximately 14 times, and the content of non-freezable bound water more than doubled. The plugging efficiency in formations with permeability of 1000~6000 millidarcy remains above 80%, and the oil recovery factor enhances by over 25% in heterogeneous formations.
(3) The strengthening mechanisms of GSNP are attributed to its adsorption onto polymer chains and its role in filling the skeletal network, thus increasing the oxidation resistance of polymers, suppressing polymer aggregation and ion penetration, and facilitating the formation of a more uniform gel structure.
(4) GSNP increases polymer hydrophilicity and relieves polymer entanglement, resulting in a more homogeneous gel network. Through the strong interactions among polymers, rigid GSNP, and water molecules, some freezable water is converted into non-freezable bound water.
(5) A proper amount of GSNP is beneficial to gel properties, while an excessive dosage can be detrimental due to reduced crosslinking density and increased particle aggregation. Careful optimization of nanoparticle size and concentration is essential to mitigate the adverse impacts of nanoparticle overfilling and aggregation.

Herein, a new method to prepare nanocomposite phenolic polymer gels with tailorable gelation and plugging properties that can meet the requirements of reservoirs with harsh conditions is proposed with low nanoparticle dosages. The novelty of this study lies in the practicable formulation of thermal and salt-tolerant gel systems with facile modified nano-silica of varying sizes and the systematic study of size and concentration effects. And the possible functional mechanisms of nano-silica of different sizes and concentrations are proposed and compared. The low viscosity, adjustable gelation time, and noticeable increase in stability and plugging performance are conducive to boosting the oil recovery factor of reservoirs with high water cut. Future work should focus on the dynamic gelation performance and migration of gelation solutions in the porous media, and the chromatographic separation of polymer and nanoparticles as well as the impacts of pore throat size should be highlighted.

## 4. Materials and Methods

### 4.1. Materials

The polyacrylamide/2-acrylamido-2-methylpropanesulfonic acid copolymer PAM-AMPS-35 (M_w_ = 20 × 10^6^ Da, AMPS content = 35%, degree of hydrolysis = 15%), provided by SNF (Taixing, China), is used as gel matrix. The representative structure of PAM-AMPS-35 with repeat units of 20 is presented in Figure S2. Hexamethylenetetramine (HMTA) and resorcinol as crosslinkers, citric acid as a pH modifier, thiourea as a deoxidizer, 3-glycidyloxypropyltrimethoxysilane (97.0%) as a nanoparticle surface modifier, and sodium chloride (NaCl), calcium chloride (CaCl_2_), magnesium chloride (MgCl_2_), and other inorganic salts for brine preparation are all purchased from Aladdin Inc., Shanghai, China. The composition of the synthetic brine is detailed in Table 3. Nano-silica sols with different particles sizes (SNP-10, SNP-30, SNP-50, and SNP-100) are obtained from Dezhou Jinghuo Technique Glass Co., Ltd., Dezhou, China. The synthetic oil used for sandpack flooding tests is prepared by mixing crude oil with kerosene, yielding a density of 0.893 g/cm^3^ at 20 °C and a viscosity of 6.28 mPa·s at 50 °C.

### 4.2. Nanoparticle Modification

Elevated temperature and/or salinity intensifies Brownian motion and compresses the diffuse electric double layer, resulting in increases particle aggregation [52,56]. To endow nanoparticles with sufficient interparticle repulsion in high-temperature and high-salinity conditions, nano-silica is functionalized with 3-glycidyloxypropyltrimethoxy silane following the previous studies [57,58]. The modified nanoparticles (denoted as GSNP) with various diameters and similar ligand coverage (~2.9 μmol/m^2^) are synthetized as follows. First, a proper amount of 3-glycidyloxypropyltrimethoxysilane is added dropwise to the diluted hydrochloric acid (pH = 2.0) under vigorous stirring at ambient temperature to facilitate the ring opening of the epoxy group (Figure S3). The pH of the solution is then adjusted to approximately 10.0 with a concentrated sodium hydroxide solution and added dropwise to the nano-silica sol (10.0 wt.% particle concentration) under vigorous stirring at 60 °C for 24 h to graft the silane onto the silica surface. Thereafter, methanol (12.5 vol.%) is added to increase silane solubility. When the reaction is completed, the methanol is evaporated, and the excess modifier and by-products are removed via dialysis. The effective nanoparticle concentration is determined using the mass–volume equation, and the nanoparticle size is measured by a dynamic light-scattering Zetasizer (Malvern, Malvern City, UK). The resulting products are labeled GSNP-10, GSNP-30, GSNP-50, and GSNP-100, respectively.

### 4.3. Preparation of Gelation Solution

The gel formulation consists of 0.5 wt.% PAM-AMPS-35, 0.35 wt.% HMTA, 0.25 wt.% resorcinol, and x wt.% nano GNP (x: 0.1–1.0). To prepare 20 g of gelation solution, 5 g of PAM-AMPS-35 polymer stock solution (2.0 wt.%) is mixed with 9.65~10.03 g of synthetic brine under stirring for 20 min. Then, 0.02~0.2 g of GSNP is added, and the mixture is homogenized by stirring and sonification. Finally, a solution containing HMTA, resorcinol, thiourea (0.07 g, 0.05 g, and 0.03 g, respectively), and 5.0 g of synthetic brine is added dropwise to the polymer/nano-silica solution under stirring at 400 rpm for 10 min. The pH of the system is adjusted to approximately 6.5 using citric acid.

The apparent viscosity of the solution is measured every 30 min with a rotational viscometer (DV2TLV, Brookfield, Brookfield, MA, USA) at a constant shear rate of 6 s^−1^. The gelation time is defined as the inflection point on the viscosity–time curve [59]. Long-term stability is tested by sealing the gelation solution in an ampoule and aging it at 90 °C. Gel strength is qualitatively evaluated according to the Sydansk gel code [60,61].

### 4.4. Microscopic Morphology Study

The morphology of the polymer gel in with or without GSNP is studied by a cryo-scanning electron microscope instrument (SU8600, Hitachi, Tokyo, Japan). Gel samples are placed in a Petri dish and are frozen in the slushed liquid nitrogen atmosphere to preserve their original microstructures. The frozen samples are transferred to a drying instrument and the excess moisture is removed by fracturing and subliming. Samples are thereafter sputtered with platinum before tests. The tests are implemented at an accelerating voltage of 5 kV with a working distance of 5–10 mm.

### 4.5. Differential Scanning Calorimetry (DSC) Tests

The enthalpy (*Q*) of freezable water phase transition in gel samples is determined by differential scanning calorimetry (DSC 3^+^, METTLER TOLEDO, Zurich, Switzerland). The instrument is preheated for 20 min prior to analysis. A sample of 10~30 mg is sealed in a hermetic pan under a nitrogen purge rate of 100 cm^3^/min. The temperature is scanned from –50 to 40 °C at a heating rate of 10 °C/min. The proportion of freezable water (*w_f_*) is calculated as the ratio of adsorbed heat of freezable water to the melting enthalpy of the bulk water (Δ*H* = 333.5 J/g). The bound water content (*w_b_*) is thereafter determined as (1-*w_f_*) [55].

### 4.6. Rheological Tests

The storage modulus (*G*′) and loss modulus (*G*″) of the gels are measured through a rotational rheometer (HAAKE Mars 40, Thermo Fisher Scientific, Waltham, MA, USA) at a constant shear stress of 1 Pa over a frequency range of 0.1~10 Hz. All measurements are performed at 25 °C and ambient pressure. Oscillation frequency sweep tests are performed using a parallel plate geometry system (diameter: 100 mm; gap: 1 mm). Zero gap and force reset procedures are implemented before each set of runs. Samples are carefully loaded and trimmed to ensure full contact. A higher storage module indicates higher gel strength [21,26].

### 4.7. Sandpack Flooding Tests

#### 4.7.1. Single Sandpack Flooding Tests

Plugging performance of gel systems is evaluated through single sandpack flooding tests. Sandpacks (2.5 cm diameter × 30 cm length) are wet packed with fresh mesh quartz sand of varying sizes to achieve different permeabilities while maintaining similar wettability. The sandpack is prepared as follows. First, the sandpack filled with synthetic brine is positioned vertically and quartz sand is added in five increments until the sandpack is completely filled. In each step, the sand is shaken slightly after being poured in. The porosity and absolute permeability of the sandpacks are 20~35% and 1000~6000 millidarcy, respectively. The properties of sandpacks are presented in Tables S2 and S3.

The displacement experiments are conducted horizontally. After the brine permeability of the sandpack is measured, a gelation solution slug of 0.3 pore volume (PV) is injected at 0.3 mL/min. The sandpack is then sealed and aged at 90 °C for 120 h. Subsequent brine flooding is thereafter conducted, and pressure is recorded every 10 min. The equilibrium pressure is used to calculate final permeability and plugging rate.

#### 4.7.2. Parallel Sandpack Flooding Tests

The enhanced oil recovery (EOR) performance of gel systems in heterogeneous formations is tested through parallel sandpack flooding tests. Sandpacks with permeabilities of 500, 1000, and 3000 millidarcy are prepared according to the method described in Section 4.7.1. The properties of sandpacks are presented in Table S4, and the schematic diagram of the experimental setup is provided in Figure S4.

After measuring the brine permeabilities, synthetic oil is injected at 0.1 mL/min until the water cut is below 1%. The oil saturation is estimated based on the volume of produced brine. Thereafter, the sandpacks are sealed and aged in a thermostat for 24 h. Water flooding is then conducted at 0.3 mL/min until the water cut exceeds 90%. A gelation solution slug of 0.3 PV is then injected, and the system is sealed and aged for 120 h. Subsequent waterflooding is conducted until the water cut reaches 90%. Pressure and production data are recorded every 10 min, and the water cut and the oil recovery factor are calculated subsequently. All experiments are performed at 90 °C.

## Figures and Tables

**Table 1:** Summary of the recent progress in silica-composite polymer gels.

Authors, Year, and References	Polymer/Crosslinker	Silica Size, nm	Functions of Nano-Silica/Performance
Maleki-Khalan et al. (2025), [42]	Sulfonated polyacrylamide (SPAM)/Chromium acetate	85~95	Enhance gel strength and swelling capacity/Oil factors increase to 57.21% in heterogeneous micromodels
Qiao, W. et al. (2023), [43]	Partially hydrolyzed polyacrylamide (HPAM)/Chromium acetate	20	Suppress polymer degradation, increase gel strength, improve network homogeneity/Plugging rate > 93%
Ashraf Soliman, A et al. (2020), [44]	Xanthan gum/Silica	<50	Increases shear resistance, thermal and salt resistance/Displacement efficiency > 80%
Amir, Z. et al. (2022), [31]	Polyacrylamide (PAM)/Polyethylenimine (PEI)	5	Increase gel strength and thermal stability/Storage modulus > 100 Pa
Liang, T. et al. (2020), [45]	α-starch/N,N’-methylene bisacrylamide	30	Increase viscosity/Oil recovery around 30%
Guo, H. et al. (2022), [46]	Partially hydrolyzed polyacrylamide (HPAM)/Water-soluble phenolic resin (WSPR)	7	Improve structure homogeneity and gel strength, inhibit polymer degradation/Storage modulus > 10 Pa, dehydration ratio < 10%
Bila, A. et al. (2021), [47]	Poly (methacrylate) based/None	32~218	Reduce IFT, emulsify oil, induce wettability alteration/Oil recovery increases by 1.51–6.13%
Telin, A. (2024), [28]	Partially hydrolyzed polyacrylamide (HPAM)/Resorcinol and paraform	7.2~69.9	Increase structural and mechanical characteristics/Residual resistance factor > 90
Mandal, M. et al. (2024), [38]	PHPA-LM and PHPA-HM/Hydroquinone (HQ) and Hexamethylenetetramine (HX)	30	Compact gel network structure/Temperature tolerance increases by over 50 °C
Liu, M. et al. (2024), [48]	Partially hydrolyzed polyacrylamide (HPAM)/Water-soluble phenolic resin (WSPR)	7	Improve the yield stress and long-term thermal stability, produce high-density cavities/Dehydration rate < 5 wt.%, plugging rate > 90%

**Table 2:** Sydansk codes of different gel systems.

Size, nm	GSNP Concentration, wt.%				
	0.2	0.4	0.6	0.8	1.0
10	D	E	E	D	C
30	E	F	F	D	D
50	E	F	F	D	D
100	D	E	E	D	C

**Table 3:** Ion compositions of the synthetic brine.

Ions	Na^+^/K^+^	Ca^2+^	Mg^2+^	Cl^−^	HCO_3_^−^	SO_4_^2−^
Concentration (mg/L)	18,327	6896	282	39,560	351	2040

## Data Availability

The data that support the findings of this study are available within the article.

## Supplementary Materials

The following supporting information can be downloaded at: https://www.mdpi.com/article/10.3390/gels11100769/s1, Figure S1: The storage modulus of polymer gels strengthened by GSNP of varying sizes at different oscillation frequencies. (a) 10 nm, (b) 30 nm, (c) 50 nm and (d) 100 nm; Figure S2: Molecular structures of (a) PAM-AMPS-35 and (b) 3-glycidyloxypropyltrimethoxysilane. Color identification: C violet; H white; O red; S yellow; N blue; Figure S3: Acidic ring opening of 3-glycidyloxypropyltrimethoxy silane; Figure S4: Schematic diagram of experimental setup of parallel sand-pack flooding test; Table S1: Properties of bare and modified nanoparticles in different solutions; Table S2: Sandpacks to study the impacts of GSNP size and concentration; Table S3 Sandpacks to study the impacts of permeability on the plugging performance of gel systems; Table S4 Sandpacks for parallel sandpack flooding tests.
