# Supplementary material for: The Size Effects of Modified Nano-Silica on the Physical Properties of Resorcinol-Poly(acrylamide-co-2-acrylamido-2-methylpropanesulfonic acid) Gels in Harsh Reservoir Conditions
Source: Gels. 2025 Sep 24;11(10):769. doi: 10.3390/gels11100769 (PMC12563236; doi:10.3390/gels11100769)
Supplement: Supplementary file 1 [file gels-11-00769-s001.zip › gels-3852618-supplementary.pdf]

Supporting Information

The Size Effects of Modified Nano-Silica on the Physical Properties of Resorcinol-Poly(Acrylamide-Co-2-Acrylamido-2-Methylpropanesulfonic Acid) Gels in Harsh Reservoir Conditions

Xun Zhong [1,2,*], Yuxuan Yang [1], Jiating Chen [3], Yudan Dong [1], Sheng Lei [1,*], Hui Zhao [1,2,4], Hong He [1,2] and Lifeng Chen [1,2]

[1] College of Petroleum Engineering, Yangtze University, Wuhan 430100, China; yangyuxuan0000@163.com (Y.Y.); 18653262028@163.com (Y.D.); zhaohui@yangtzeu.edu.cn (H.Z.); hehong1103@163.com (H.H.); lyj505523522@126.com (L.C.)
[2] Key Laboratory of Drilling and Production Engineering for Oil and Gas, Wuhan 430100, China
[3] Shunan Gas Mine, Southwest Oil and Gas Field Branch, China National Petroleum Corporation, Luzhou 646000, China; chenjiating@petrochina.com.cn
[4] Western Research Institute, Yangtze University, Karamay 834000, China
* Correspondence: zhongxun@yangtzeu.edu.cn (X.Z.); lei950921@163.com (S.L.)

Table S1. Properties of bare and modified nanoparticles in different solutions.

Properties / Nanoparticle	Medium diameter，nm				Zeta potential (in distilled water)，mV	
	Bare particle		Modified particle		Bare particle	Modified particle
	DI water	Brine	DI water	Brine		
SNP-10	10.4±2.2 (PDI=0.12)	Turbidity	12.6±1.7 (PDI=0.20)	13.2±1.4	-35.3±2.5	-18.5±3.7
SNP-30	32.1±1.5 (PDI=0.15)	Turbidity	28.5±2.2 (PDI=0.24)	31.6±1.8	-30.4±1.6	-21.1±2.2
SNP-50	56.0±7.9 (PDI=0.22)	Precipitate	49.2±6.3 (PDI=0.31)	52.3±3.7	-26.7±2.1	-17.9±1.5
SNP-100	110.0±16.6 (PDI=0.34)	Precipitate	125.0±8.9 (PDI=0.36)	122.0±5.4	-20.8±3.5	-16.3±0.8

Table S2. Sandpacks to study the impacts of GSNP size and concentration.

No.	Permeability (mD)	Porosity (%)	Specifications
S1	994	21.43	Baseline
S2	1021	20.55	0.4% wt. GSNP-10
S3	981	21.36	0.4% wt. GSNP-30
S4	995	21.07	0.4% wt. GSNP-50
S5	946	20.43	0.4% wt. GSNP-100
S6	1055	20.19	0.2% wt. GSNP-30
S7	1037	20.43	0.6% wt. GSNP-30
S8	1026	20.67	0.8% wt. GSNP-30
S9	953	21.66	1% wt. GSNP-30

Table S3. Sandpacks to study the impacts of permeability on the plugging performance of gel systems.

No.	Permeability(mD)	Porosity (%)	Specifications
S10	1989	24.13	Baseline
S11	2000	24.09	0.4% wt. GSNP-30
S12	2005	23.84	0.4% wt. GSNP-100
S13	2954	27.66	Baseline
S14	3021	28.42	0.4% wt. GSNP-30
S15	3009	27.89	0.4% wt. GSNP-100
S16	4078	31.88	Baseline
S17	4022	32.05	0.4% wt. GSNP-30
S18	3976	31.26	0.4% wt. GSNP-100
S19	5896	34.77	Baseline
S20	6105	35.12	0.4% wt. GSNP-30
S21	6083	34.63	0.4% wt. GSNP-100

Table S4. Sandpacks for parallel sandpack flooding tests.

No.	Permeability ratio	Permeability (mD)	Porosity (%)
S22	2:1	2011	23.88
S23		1022	20.87
S24	6:1	6021	33.54
S25		974	21.62

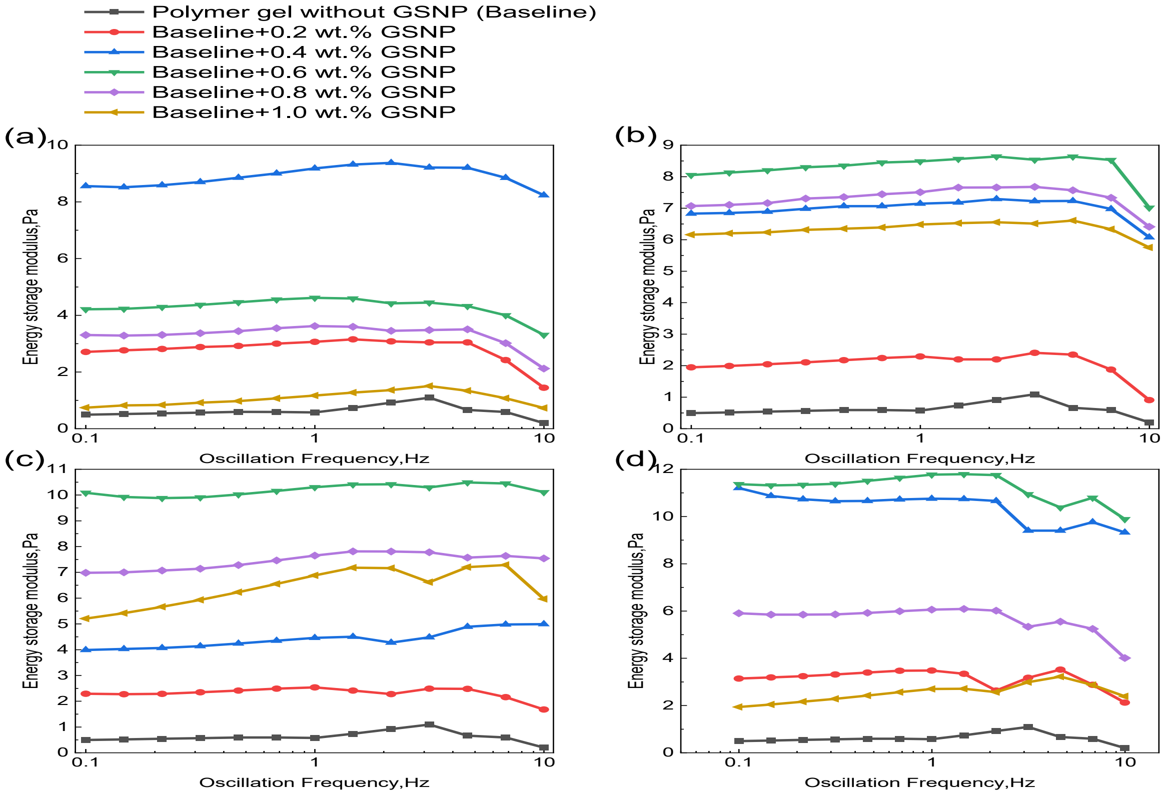

Figure S1. The storage modulus of polymer gels strengthened by GSNP of varying sizes at different oscillation frequencies. **(a)** 10 nm, **(b)** 30 nm, **(c)** 50 nm and **(d)** 100 nm.

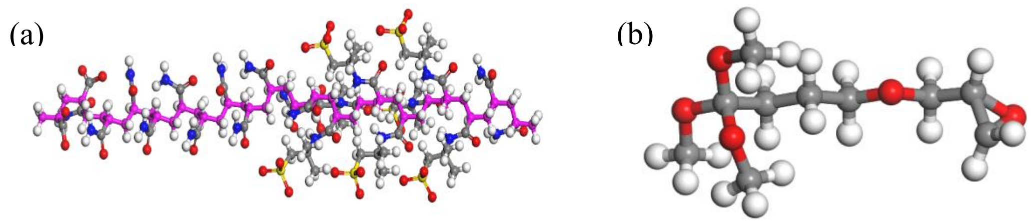

Figure S2. Molecular structures of **(a)** PAM-AMPS-35 and **(b)** 3-glycidyloxypropyltrimethoxysilane. Color identification: C violet; H white; O red; S yellow; N blue.

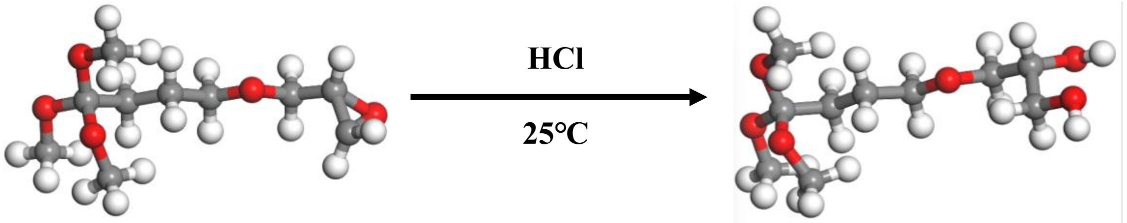

Figure S3. Acidic ring opening of 3-glycidyloxypropyltrimethoxy silane.

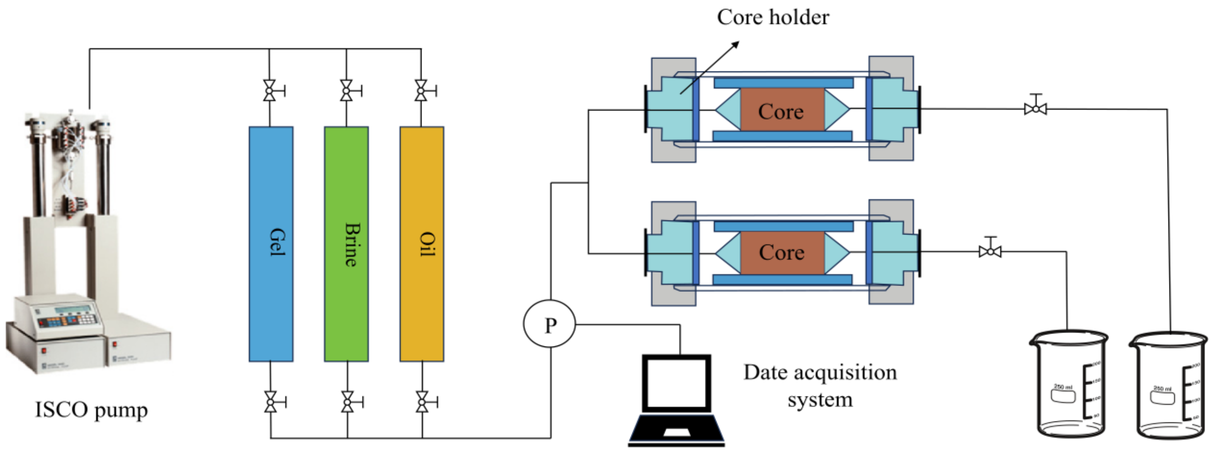

Figure S4. Schematic diagram of experimental setup of parallel sand-pack flooding test.
